# Deletion allele of Apo B gene is associated with higher inflammation, oxidative stress and dyslipidemia in obese type 2 diabetic patients: an analytical cross-sectional study

**DOI:** 10.1186/s12902-022-00991-y

**Published:** 2022-03-22

**Authors:** Nasim Mokhtary, Seyedeh Neda Mousavi, Gity Sotoudeh, Mostafa Qorbani, Maryam Dehghani, Fariba Koohdani

**Affiliations:** 1 Department of Cellular and Molecular Nutrition, School of Nutritional Sciences and Dietetics, Tehran University of Medical Sciences, Tehran, Iran; 2 Metabolic Diseases Research Center, Zanjan University of Medical Sciences, Zanjan, Iran; 3 Department of Nutrition, School of Medicine, Zanjan University of Medical Sciences, Zanjan, Iran; 4 Department of Community Nutrition, School of Nutritional Sciences and Dietetics, Tehran University of Medical Sciences, Tehran, Iran; 5 Non-Communicable Diseases Research Center, Alborz University of Medical Sciences, Karaj, Iran; 6 Prevention of Metabolic Disorders Research Center, Research Institute for Endocrine Sciences, Shahid Beheshti University of Medical Sciences, Tehran, Iran; 7 Diabetes Research Center, Endocrinology and Metabolism Clinical Sciences Institute, Tehran University of Medical Sciences, Tehran, Iran

**Keywords:** Inflammation, Oxidative stress, Obesity, *ApoB* gene, Polymorphism

## Abstract

**Background:**

We decided to compare some inflammatory, and oxidative stress markers, as well as lipid profiles between the obese and non-obese patients with type 2 diabetes considering ApoB gene polymorphism.

**Methods:**

one-hundred sixty two patients with type 2 diabetes were included in this study. ApoB genotyping was conducted by the polymerase chain reaction. Serum interleukin-(IL-18), pentraxin-3 (PTX-3), and high sensitive- C reactive protein (hs-CRP) was measured as the inflammatory markers. Moreover, copper-zinc superoxide dismutase (Cu/Zn-SOD), total antioxidant capacity (TAC) and 8-isoprostane F2α were analyzed for oxidative stress assessment. Anthropometric indices and lipid profiles were measured.

**Results:**

Adjusted for confounders, serum hs-CRP (*p* = 0.04), LDL-C (*p* = 0.01), LDL-C/HDL-C (*p* = 0.04), and TG (*p* = 0.02) were significantly lower at the Homozygous Insertion (Ins)/Ins *vs.* deletion (Del) allele carriers in the obese patients. Serum TAC was significantly lower at the obese Del allele carriers than Ins/Ins Homozygous (*p* = 0.03). Serum hs-CRP (*p* = 0.006), and 8-IsoprostanF2α (*P* = 0.04) were significantly higher in the obese Del allele carriers than non-obese. Serum Cu/Zn-SOD was significantly higher in the non-obese Del allele carriers than obese (*p* = 0.04).

**Conclusion:**

Inflammation, dyslipidemia, and oxidative stress are higher in the Obese Del allele carriers with type 2 diabetes which prone them to other chronic disorders.

## Introduction

Cardiovascular diseases (CVDs) are one of the main cause of morbidity and mortality in the world [1]. CVDs are a chronic complex disorder with strong genetic and environmental risk factors. It is well determined that dyslipidemia is an independent risk factor for CVDs [2]. Patients with type 2 diabetes (T2DM) are more susceptible to CVDs [3]. Obesity, especially visceral fat, is the major environmental risk factor for T2DM. Excess calorie intake leads to increase in size and number of adipocytes, which result in macrophages accumulation in adipose tissues. Inflammatory markers released from these resident cells induces low grade inflammatory state in the body which finally lead to chronic diseases occurrence [4]. Inflammatory conditions disturb the oxidant and antioxidant status in the body and create oxidative stress [5, 6]. Inflammation, oxidative stress and dyslipidemia are the main causes of CVDs in the type 2 diabetic patients [7, 8].

Apolipoprotein B (ApoB) is the main apoprotein on LDL, very low-density lipoprotein (VLDL), intermediate-density lipoprotein (IDL) and lipoprotein (a) particles [9]. Despite to the role of ApoB in dyslipidemia and CVDs [10], we found inconsistent results across various studies. In addition, the association between the existence of deletion (Del) allele on ApoB gene with serum inflammatory and oxidative stress markers in type 2 diabetic patients still is not studied up to date. It is possible that different ApoB genotypes could have various effects on the inflammation and oxidative stress. Therefore, we decided to answer the question that is Del allele associated with higher serum inflammatory and oxidative stress markers in type 2 diabetic patients? Moreover, is this association similar in obese and non-obese patients?

## Materials and methods

### Study design and participants

An analytical cross-sectional study was conducted to compare some inflammation, and oxidative stress markers, as well as lipid profile in type 2 diabetic patients with Ins/Ins Homozygous vs. Del allele carriers of ApoB gene. Totally, 162 patients (59 male and 103 female), aged 35–65 yrs. were enrolled. Exclusion criteria were as follows: pregnancy and/or lactating, insulin therapy and alcohol consumption 24 h. before the study enrollment. All of patients signed the consent form. All methods were performed in accordance with the cross-sectional study guidelines.

### Patient’s characteristics, anthropometrics and physical activity level

Basal characteristics were recorded at the interview. Weight and height were measured according to the standards by the Seca scale. Waist circumference (WC) was measured at the midpoint between the upper and lower edge of the iliac crest and the last rib. Measurements were taken by a dietitian. BMI (Body Mass Index) was calculated according to the formula: BMI = weight/ height2 (kg/m2). Then, participants were categorized to the obese (BMI ≥ 30 kg/m2) and non-obese (BMI < 30 kg/m2) according to the WHO criteria [11]. Physical activity (PA) was assessed by the classified PA-questionnaire according to metabolic equivalent task (Met).

### Dietary intake

Face-to-face interview was done by a trained dictation for gathering patient’s dietary intake during the last year. A semi-quantitative food frequency questionnaire (FFQ) for 148 food items was used for this purpose which was validated previously [12]. The nutritionist 3 (N3) software was used to analyze the macronutrients and energy intake.

### Biochemical measures

Fasting venous blood samples were taken at 7:00- 8:00 AM. Serum total cholesterol (TC) and triglyceride (TG) were measured through an enzymatic method by standard kit (Pars Azmoon kit, Tehran, Iran). Low-density lipoprotein-cholesterol (LDL-C-) and high-density lipoprotein-cholesterol (HDL-C) were measured through turbidimetry method (Hitachi, Roche Co., Germany). Serum Interleukin 18 (IL-18), pentraxin 3 (PTX3) and 8-isoprostane F2α were measured by Crystal Day Biotech kit (Shanghai Crystal Day Biotech Co, China) and DBC kit (Diagnostics Biochem Canada Co, Canada) was used for the hs-CRP measurement. Total antioxidant capacity (TAC) and copper–zinc superoxide dismutase (Cu/Zn-SOD) were measured by spectrophotometry and colorimetery methods, respectively.

### Genotyping

Firstly, genomic DNA was extracted from whole blood by salting out method [13]. Genotyping of ApoB Ins/Del obtained by using PCR (polymerase chain reaction) and PAGE (polyacrylamide gel electrophoresis). The forward and reverse primers were as follows, respectively: 5’CAGCTGGCGATGGACCCGCCGA3’ and 3’ACCGGCCCTGGCGCCCGCCAGCA5’. The Bioneer Co. synthesized primers. 25 μl mixture for PCR was provided by combination of 2.5 mM of each forward and reverse primers, 50 ng DNA, 2xTaq polymerase mix, 7% dimethyl sulfoxide. 5 μg of PCR products combined with 3 μg of BFBM (bromophenol blue). That mixture (8 μg) was put on 8% polyacrylamide gel. Electrophoresis was done.

### Statistical analysis

Normal distribution of continuous variables was checked by the kolmogrov-smirnov test. Then, logarithmic transformations were used for non-normal variables. Quantitative and qualitative variables were analyzed by the independent sample t-test and chi-square test, respectively. ANCOVA test was used for adjusting the confounders including lipid-lowering drugs, WC, carbohydrate, PUFA, MUFA. The significant level was considered as *p* < 0.05. All analyses were done by SPSS software (SPSS Inc., Chicago, IL, USA, version 16). The three-identified genotypes were categorized as Homozygous for the Insertion allele (Ins/Ins) and Del allele carriers (Ins/Del and Del/Del).

## Results

Mean (SD) age of participants was 54.19 (6.47), and 63.6% (*N* = 103) of them were women.

Table 1 shows the characteristics of participants in total population which is separately illustrated in non-obese and obese participants. Intake of lipid lowering drugs were different between the homozygous for Ins allele and Del allele carriers at the total population (*p* = 0.03). WC was nearly different between the two groups in the obese people (*p* = 0.05). Other variables had no significant difference between the Ins/Ins genotypes and Del allele carriers in the total population, as well as separately in the non-obese and obese population. (Table 1).

**Table 1:** Baseline characteristics and dietary intake of various Apolipoprotein B gene polymorphisms in patients with type 2 diabetes

Variable	**Patients**					
	Non-obese *n* = 90		Obese *n* = 72		Total *n* = 162	
Polymorphisms	Ins/Ins *n *= 60	Del carrier *n* = 30	Ins/Ins *n* = 47	Del carrier *n* = 25	Ins/Ins *n* = 107	Del carrier *n* = 55
Age, year	54.77 ± 6.25	55.73 ± 5.33	53.13 ± 6.91	52.92 ± 7.18	54.05 ± 6.57	54.45 ± 6.34
BMI, kg/m^2^	26.22 ± 2.26	26.21 ± 2.44	33.1 ± 3.57	33.29 ± 3.33	29.23 ± 4.48	29.43 ± 4.56
Waist circumference, cm^†^	86.92 ± 7.76	84.79 ± 9.02	100.34 ± 9.34	95.74 ± 9.94	92.81 ± 10.77	89.86 ± 10.87
Physical activity, time/day	39.64 ± 6.4	38.42 ± 5.3	37.55 ± 5.46	37.48 ± 3.69	38.72 ± 6.07	37.99 ± 4.62
Sex						
Male	27 (45)	10(33.3)	14 (29.8)	8 (32)	41(38.3)	18 (32.7)
Female	33 (55)	20 (66.7)	33 (70.2)	17(68)	66 (61.7)	37(67.3)
Dietary intake						
Energy, kcal/day	2572 ± 905	2680 ± 1677	2374 ± 702	2737 ± 1141	2485 ± 824	2706 ± 1445
Carbohydrate, g/day	352.97 ± 6.92	367.92 ± 9.79	318.42 ± 11	346.47 ± 15.1	338.22 ± 6.20	357.35 ± 8.66
Protein, g/day	90.52 ± 2.3	96.98 ± 3.26	85.39 ± 2.25	83.02 ± 3.11	88.61 ± 1.73	89.97 ± 2.42
Fat, g/day	101.09 ± 3.48	92.29 ± 4.92	106.52 ± 4.29	96.8 ± 5.92	103 ± 2.79	95.3 ± 3.89
PUFA, g/day	23.51 ± 1.29	21.68 ± 1.73	27.47 ± 1.53	22.97 ± 2.12	25.08 ± 1.02	22.6 ± 1.43
MUFA, g/day	32.8 ± 1.34	31.64 ± 1.89	38.03 ± 1.83	32.44 ± 2.52	34.88 ± 1.14	32.37 ± 1.6
SFA, g/day	26.51 ± 0.86	26.73 ± 1.22	27.28 ± 1.2	25.63 ± 1.66	26.76 ± 25.34	26.4 ± 1
Fiber, g/day	42.82 ± 20.65	48.95 ± 13.5	36.77 ± 15.3	42.86 ± 19.5	40.16 ± 18.7	46.18 ± 39.7
Intake of lipid lowering drugs^‡^						
Yes	29(48.3)	20 (66.7)	20 (42.6)	15 (60)	49 (45.8)	35 (63.6)
No	31(51.7)	10(33.3)	27 (57.4)	10 (40)	58 (54.2)	20 (36.4)
Intake of antidiabetic drugs						
Yes	54 (90)	27 (90)	44 (93.6)	24 (96)	98 (91.6)	51 (92.7)
No	6 (10)	3 (10)	3 (6.4)	1 (24)	9 (8.4)	4 (7.3)

^†^Significant difference in the obese population using the independent t-test (*p*<0.05)

^‡^Significant difference in total population using the chi-square test (*p*<0.05)

*BMI* body mass index, *PUFA* poly unsaturated fatty acid, *MUFA* mono unsaturated fatty acid, *SFA* saturated fatty acids

Table 2 shows the crude mean (SD) of the studied inflammatory and oxidative stress markers, as well as lipid profiles in the total population and obese/non-obese patients according to the polymorphisms. In total population, serum LDL-C (*p* = 0.04) was significantly higher in the Del allele carriers after adjusting for confounders. In obese patients and after adjusting for confounders, serum hs-CRP (*p* = 0.04), LDL-C (*p* = 0.01), LDL-C/HDL-C (*p* = 0.04) and TG (*p* = 0.02) were significantly higher in Del allele carriers than Ins/Ins Homozygous. Unlikely, serum TAC was significantly higher in the obese patients with Ins/Ins genotype than Del allele carriers (*p* = 0.03). In non-obese patients, no significant difference was shown between the Ins/Ins Homozygous vs. Del allele carriers about inflammatory and oxidative stress markers, as well as lipid profiles (Table 2). However, no significant difference was shown in the assessed variables between the obese and non-obese patients with Ins/Ins polymorphism, the Del allele significantly increased serum hs-CRP (*p* = 0.006) and 8-isoprostane F2α (*p* = 0.04) in obese vs. non-obese patients, adjusted for the confounders. Interestingly, serum Cu/Zn-SOD was significantly lower in the obese Del allele carriers than non-obese patients (*p* = 0.04). (Table 3).

**Table 2:** Inflammatory indices, oxidative stress markers and lipid profile between Ins/Ins Homozygous *vs*. Del allele carriers

Variable	**Patients**					
	Non-obese *n* = 90		Obese *n* = 72		Total *n* = 162	
Polymorphisms	Ins/Ins *n* = 60	Del carrier *n* = 30	Ins/Ins *n* = 47	Del carrier *n* = 25	Ins/Ins *n* = 107	Del carrier *n* = 55
IL-18, pg/ml	240.65 ± 1.11	247.11 ± 1.10	254.44 ± 1.12	255.38 ± 1.12	246.60 ± 1.12	250.84 ± 1.11
PTX3, ng/ml	2.71 ± 1.17	2.54 ± 1.27	2.50 ± 1.22	2.56 ± 1.19	2.61 ± 1.20	2.55 ± 1.24
hs-CRP, mg/ml	1.33 ± 3.20	1.13 ± 2.92	1.80 ± 2.66 ^**‡**^	2.52 ± 1.77	1.51 ± 2.98	1.64 ± 2.61
Cu/Zn SOD, U/ml	0.14 ± 1.37	0.15 ± 1.36	0.13 ± 1.49	0.12 ± 1.31	0.14 ± 1.43	0.13 ± 1.36
TAC, g/dl	2.55 ± 1.26	2.39 ± 1.26	2.36 ± 1.19^**‡**^	2.21 ± 1.16	2.46 ± 1.23	2.30 ± 1.22
8-IF2α, pg/ml	72.01 ± 1.09	69.77 ± 1.08	73.01 ± 1.09	74.25 ± 1.06	72.44 ± 1.09	71.77 ± 1.08
HDL.C, mg/dl	50.01 ± 1.23	54.52 ± 1.22	51.68 ± 1.22	54.45 ± 1.27	50.74 ± 1.23 ^**†**^	54.48 ± 1.24
LDL.C, mg/dl	111.11 ± 40.7	110.30 ± 29.45	105.91 ± 29.82^**†**^^**,**^^**‡**^	125.48 ± 47.83	108.83 ± 36.3 ^**‡**^	117.20 ± 39.25
LDL.C/HDL.C ratio	2.19 ± 0.75	2.05 ± 0.65	2.09 ± 0.68 ^**‡**^	2.25 ± 0.58	2.15 ± 0.72	2.14 ± 0.62
TC, mg/dl	197.51 ± 1.170	195.52 ± 1.55	188.75 ± 1.42	194.80 ± 1.52	193.64 ± 1.58	195.20 ± 1.53
TG, mg/dl	147.94 ± 1.22	141.67 ± 1.83	138 ± 1.69^**†, ‡**^	191.7 ± 1.76	143.44 ± 1.70	162.96 ± 1.82

^†^Significant difference between the two polymorphisms in the crude model;^‡^ Significant difference between the two polymorphisms adjusted for baseline parameters

*IL-18* interleukin-18, *PTX* pentrexin-3, *hs-CRP* high sensitivity-C reactive protein, *CU/ZN SOD* superoxide dismutase, *TAC* total antioxidant capacity, *8-IF2α* 8-isoprostane F2α, *HDL-C* high density lipoprotein. Cholesterol, *LDL-C* low density lipoprotein. Cholesterol, *TG* triglyceride, *TC* total cholesterol

Data are presented as mean (± SD)

**Table 3:** Inflammatory indices, oxidative stress markers and lipid profile in obese *vs*. non-obese patients with type 2 diabetes

Variable	**Polymorphisms**					
	Ins/Ins *n* = 107		*p* value	Del carriers *n* = 55		*p* value
Patients	Non-Obese *n* = 60	obese *n* = 47		Obese *n* = 25	Non-obese *n* = 30	
IL-18, pg/ml	240.65 ± 1.11	254.44 ± 1.12	**0.01**	255.38 ± 1.12	247.11 ± 1.10	0.27
PTX3, ng/ml	2.71 ± 1.17	2.50 ± 1.22	**0.02**	2.56 ± 1.19	2.54 ± 1.27	0.89
hs-CRP, mg/ml	1.33 ± 3.20	1.80 ± 2.66	0.15	2.52 ± 1.77	1.13 ± 2.92	**0.001**
Cu/Zn SOD, U/ml	0.14 ± 1.37	0.13 ± 1.49	**0.04**	0.12 ± 1.31	0.15 ± 1.36	**0.005**
TAC, g/dl	2.55 ± 1.26	2.36 ± 1.19	0.06	2.21 ± 1.16	2.39 ± 1.26	0.14
8-IF2α, pg/ml	72.01 ± 1.09	73.01 ± 1.09	0.43	74.25 ± 1.06	69.77 ± 1.08	**0.004**
HDL.C, mg/dl	50.01 ± 1.23	51.68 ± 1.22	0.41	54.45 ± 1.27	54.52 ± 1.22	0.98
LDL.C, mg/dl	111.11 ± 40.71	105.91 ± 29.82	0.46	125.48 ± 47.83	110.30 ± 29.45	0.15
LDL.C/HDL.C ratio	2.19 ± 0.75	2.09 ± 0.68	0.44	2.25 ± 0.58	2.05 ± 0.65	0.24
TC, mg/dl	197.51 ± 1.42	188.75 ± 1.70	0.61	194.80 ± 1.52	195.52 ± 1.55	0.97
TG, mg/dl	147.94 ± 1.70	138 ± 1.69	0.51	191.7 ± 1.76	141.67 ± 1.83	0.06

Data are expressed as mean± SD. Comparisons were analyzed by the independent sample t-test. *p*<0.05 is significant

## Discussion

In the present study, we found that Del allele of ApoB gene not only is associated with higher serum LDL-C, LDL-C/HDL-C ratio, hs-CRP, and 8-isoprostane F2α, but also lower serum TAC and Cu/Zn-SOD was shown in this group. Serum TAC was significantly decreased in the obese Del allele carriers than non-obese type 2 diabetic patients. Serum LDL-C and LDL-C/HDL-C ratio were higher in the obese Del allele carriers than non-obese. In the obese diabetic patients, serum LDL-C and LDL-C/HDL-C ratio were significantly higher in the Del allele carriers than Ins/Ins Homozygous. All of these alterations predispose the obese type 2 diabetic patients to the CVDs.

Apo-lipoproteins are proteins that combine with lipids to regulate their metabolism. There are various isotypes of apo-lipoproteins (Apo A (I, II, IV), B, C (I, II, III), and E) [14]. Apolipoprotein B (ApoB) is a key component of all the atherogenic lipoproteins including LDL, VLDL, IDL, and lipoprotein (a) that plays a major role in the cholesterol balance [9]. It is approved that ApoB is superior to the LDL-C in prediction of CVDs. There are various polymorphisms on ApoB gene such as XbaI, EcoRI, SpIns/Del [15]. Despite the small sample size of the most previous studies, findings propose the significant role of Ins/Del polymorphisms on risk of CVDs, as well as myocardial infarction (MI) [16]. ApoB is an important risk factor for atherosclerosis and MI because of its association with atherogenic lipid profiles including LDL-C, VLDL and chylomicron remnants. The relation between Del allele of ApoB gene and MI has been proved in the previous meta-analysis [17]. Previous studies on the association between the Del allele of ApoB gene and coronary heart disease (CHD) had inconclusive results. At the Kuwaiti participants, no association was shown between Del allele and dyslipidemia, as well as hypertension. However, Del allele Homozygous for ApoB gene had significant odd ratio for CHD (OR = 2.43, CI = 1.34–4.41) [18]. In diabetic population, significant association was reported between Del allele and dyslipidemia, as well as general obesity [9]. In the Indian population, Del allele carriers had more BMI. Serum lipid profile had no significant difference between Ins/Ins Homozygous and Del allele carriers [19]. Homozygous women for Ins allele had lower TC, LDL-C and ApoB levels at the Czech population [20]. Another study showed the significant association between Apo A2-256 T/C polymorphism with inflammatory indices and oxidative stress markers. The T allele of Apo A2-256 T/C polymorphism had protective effect against oxidative stress and inflammation [21, 22].

Dyslipidemia, inflammation and oxidative stress are the main causes of chronic complications of type 2 diabetes such as CVDs. Obesity is a most important cause of type 2 diabetes [23, 24]. Inflammatory indices and oxidative stress are markedly higher in the obese type 2 diabetic patients. More IL-6 and hs-CRP are produced by the liver, which leads to insulin resistance and endothelial dysfunction [25].

Oxidative stress is a situation of imbalance between the oxidant and antioxidant system. However, there is various enzymatic (superoxide dismutase, catalase, glutathione peroxidase etc.) and non-enzymatic (carotenoids, tocopherols, ascorbate, bioflavonoids, bilirubin, uric acid etc.) antioxidants in the serum, diabetic patients have defect in antioxidant system and cannot scavenge the produced-ROS (superoxide anion, hydroxyl radical, hydrogen peroxide etc.) which has pathological effects [26]. TAC describes the cumulative effect of all antioxidants present in serum and fluids [27]. 8-isoprostane F2α is an independent risk factor for coronary heart disease incidence in diabetic patients [28]. Previous studies reported increase in 8-isoprostane F2α in diabetic patients, patients with dyslipidemia, hypertension and smokers [29–31]. Moreover, the relationship between 8-isoprostane F2α and serum hs-CRP has been reported [28]. Our results showed that serum hs-CRP and 8-IsoprostanF2α were significantly higher in the obese Del allele carriers than non-obese. Serum Cu/Zn-SOD was significantly higher in the non-obese Del allele carriers than obese. This means that serum antioxidant capacity decreases and inflammation increases in the obese Del allele carriers, which predispose this group to the oxidative stress and inflammatory conditions and finally CVDs.

No association was shown between IL-18 and ApoB polymorphism. In a previous study, gene expression of IL-18 was up regulated due to insulin resistance. Moreover, IL-18 showed significant association with anthropometric indices including BMI, WC, and WHtR in all participants, but not in the diabetic patients [32], which is in agree with our result.

The significant effect of Del allele was seen on some of inflammatory indices and oxidative stress markers in the obese type 2 diabetic patients. We propose that future studies with more sample size must be conducted on healthy people or patients with CVD.

In conclusion, Iranian obese patients with type 2 diabetes that are Del allele carrier in *Apo B* gene, are more susceptible to cardiovascular diseases because of more inflammation, dyslipidemia and oxidative stress in the body. Identification and screening of these patients for *Apo B* gene polymorphism can help these patients from other chronic diseases.

## Data Availability

The datasets used and/or analyzed during the current study available from the corresponding author on reasonable request.

## References

[1] Alalawi, H.H. and S.A. MANAL, Detection of Cardiovascular Disease using Machine Learning Classification Models*.* INTERNATIONAL JOURNAL OF ENGINEERING RESEARCH & TECHNOLOGY (IJERT) ISSN: p. 2278–0181.

[2] Cruz-Bautista I (2015). Determinants of VLDL composition and apo B-containing particles in familial combined hyperlipidemia. Clin Chim Acta.

[3] Martín-Timón I (2014). Type 2 diabetes and cardiovascular disease: have all risk factors the same strength?. World J Diabetes.

[4] Chadt, A., et al., Molecular links between obesity and diabetes:“diabesity”*.* Endotext [Internet], 2018.

[5] Fernández-Sánchez A (2011). Inflammation, oxidative stress, and obesity. Int J Mol Sci.

[6] Kattoor AJ (2017). Oxidative stress in atherosclerosis. Curr Atheroscler Rep.

[7] Tangvarasittichai S (2015). Oxidative stress, insulin resistance, dyslipidemia and type 2 diabetes mellitus. World J Diabetes.

[8] Holvoet P (2008). Relations between metabolic syndrome, oxidative stress and inflammation and cardiovascular disease. Verh K Acad Geneeskd Belg.

[9] Rafiee M (2016). Association between Insertion/Deletion Polymorphism of ApoB Gene with Dyslipidemia and Obesity Risk in Patients with Type 2 Diabetes. J Obes Overweig.

[10] Barter PJ (2006). Apo B versus cholesterol in estimating cardiovascular risk and in guiding therapy: report of the thirty-person/ten-country panel. J Intern Med.

[11] Organization, W.H., Obesity: preventing and managing the global epidemic*.* 2000. 11234459

[12] Mirmiran P (2010). Reliability and relative validity of an FFQ for nutrients in the Tehran lipid and glucose study. Public Health Nutr.

[13] MWer S , Dykes D, Polesky H (1988). A simple salting out procedure for extracting DNA from human nucleated cells. Nucleic acids res.

[14] Mahley RW (1984). Plasma lipoproteins: apolipoprotein structure and function. J Lipid Res.

[15] Tsunoda K (2012). Polymorphism of the apolipoprotein B gene and association with plasma lipid and lipoprotein levels in the Mongolian Buryat. Biochem Genet.

[16] Chiodini BD (2003). APO B gene polymorphisms and coronary artery disease: a meta-analysis. Atherosclerosis.

[17] Li Y-Y (2014). ApoB gene SpIns/Del, XbaI polymorphisms and myocardial infarction: a meta-analysis of 7169 participants. J Cardiovasc Med.

[18] Al-Bustan, S.A., et al.,Increased Risk of the APOB rs11279109 Polymorphism for CHD among the Kuwaiti Population*.* Disease markers, 2017. 2017. 10.1155/2017/6963437 PMC5737435 29362515

[19] Saha N (1993). DNA polymorphisms of the apolipoprotein B gene are associated with obesity and serum lipids in healthy Indians in Singapore. Clin Genet.

[20] Hubacek JA (2001). Genetic determination of plasma lipids and insulin in the Czech population. Clin Biochem.

[21] Koohdani F (2015). Association between ApoA-II-265 T/C polymorphism and oxidative stress in patients with type 2 diabetes mellitus. J Diabetes Complications.

[22] Koohdani F (2016). APO A2–265T/C polymorphism is associated with increased inflammatory responses in patients with type 2 diabetes mellitus. Diabetes Metab J.

[23] Arrieta  F (2018). Diabetes mellitus Y riesgo cardiovascular. Actualización de las recomendaciones del Grupo de Trabajo de diabetes Y Riesgo cardiovascular de la Sociedad Española de diabetes (SED, 2018). Clínica e Investigación en Arteriosclerosis.

[24] Lotfy M (2017). Chronic complications of diabetes mellitus: a mini review. Curr Diabetes Rev.

[25] Piva SJ (2013). Assessment of inflammatory and oxidative biomarkers in obesity and their associations with body mass index. Inflammation.

[26] Delmas-Beauvieux M-C (1996). The enzymatic antioxidant system in blood and glutathione status in human immunodeficiency virus (HIV)-infected patients: effects of supplementation with selenium or beta-carotene. Am J Clin Nutr.

[27] Kampa M (2002). A new automated method for the determination of the Total Antioxidant Capacity (TAC) of human plasma, based on the crocin bleaching assay. BMC Clin Pathol.

[28] Schwedhelm E (2004). Urinary 8-iso-prostaglandin F2α as a risk marker in patients with coronary heart disease: a matched case-control study. Circulation.

[29] Davì  G (1999). In vivo formation of 8-iso-prostaglandin F2α and platelet activation in diabetes mellitus: effects of improved metabolic control and vitamin E supplementation*.*. Circulation.

[30] Minuz P (2002). Increased oxidative stress and platelet activation in patients with hypertension and renovascular disease. Circulation.

[31] Morrow JD (1995). Increase in circulating products of lipid peroxidation (F2-isoprostanes) in smokers—smoking as a cause of oxidative damage. N Engl J Med.

[32] Zirlik A (2007). Interleukin-18, the metabolic syndrome, and subclinical atherosclerosis: results from the Dallas Heart Study. Arterioscler Thromb Vasc Biol.

